# *ifas-1* is upregulated by fungal infection in a GPA-12 and STA-2-independent manner in the *Caenorhabditis elegans* epidermis

**DOI:** 10.17912/micropub.biology.000400

**Published:** 2021-05-25

**Authors:** Shizue Omi, Xing Zhang, Nishant Thakur, Nathalie Pujol

**Affiliations:** 1 Aix Marseille Univ, INSERM, CNRS, CIML, Turing Centre for Living Systems, Marseille, France

## Abstract

Skin infection with the fungus *Drechmeria coniospora* leads to a transcriptional response in the worm epidermis. This involves an increased expression of a group of antimicrobial peptide (AMP) genes including those in the *nlp-29* and *cnc-2* clusters. The major pathways leading to the expression of these AMP genes have been well characterized and converge on the STAT transcription factor STA-2. We reported previously that expression in the epidermis of a constitutively active (gain of function, gf) form of the Gα protein GPA-12 (GPA-12gf) recapitulates much of the response to infection. To reveal parallel pathways activated by infection, we focus here on an effector gene that is not induced by GPA-12gf. This gene, *ifas-1*, encodes a protein with a fascin domain, associated with actin binding. Its induction upon fungal infection does not require *sta-2*. A transcriptional reporter revealed induction in the epidermis of *ifas-1* by infection and wounding. Thus, *ifas-1* represents part of a previously unexplored aspect of the innate immune response to infection.

**Figure 1. Induction of ifas-1 expression upon infection is independent of STA-2:**
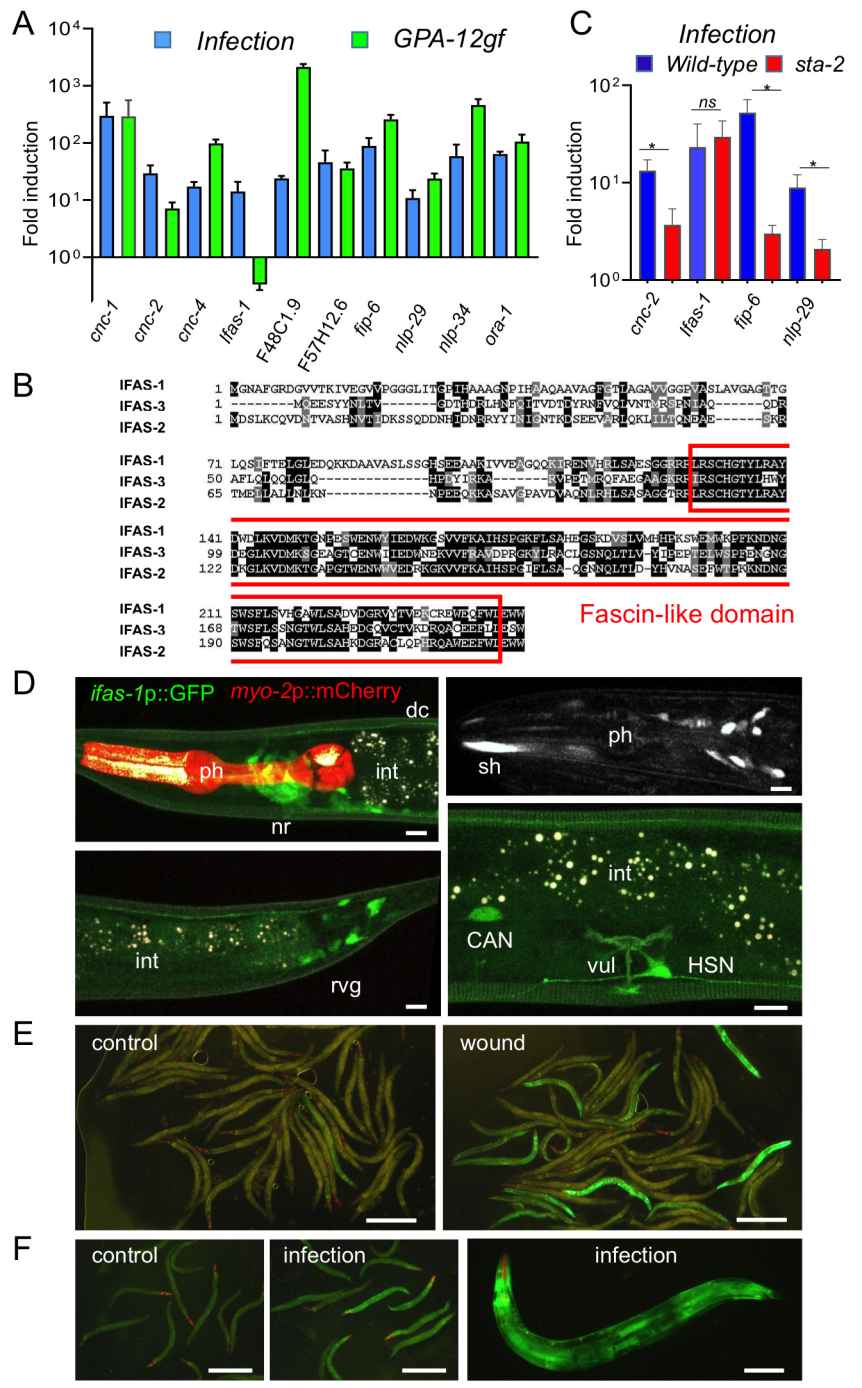
A) Quantitative RT-PCR analysis of the expression of 10 genes that were reported to be induced by *D. coniospora* infection in RNAseq experiments. Levels of gene expression in wild type worms after 8 h of infection or in a strain expressing GPA-12gf under the control of a promoter active in the adult epidermis were compared to age-matched uninfected wild-type worms, mean results with SEM from 3 independent experiments are shown. B) Sequence alignment of IFAS-1, IFAS-2 and IFAS-3 proteins; the fascin domain is boxed in red. C) Quantitative RT-PCR analysis of the change in expression of 4 genes in wild type (blue) or *sta-2* mutant worms (red) after 8 h of infection compared to age-matched uninfected worms. Mean results with SEM from 3 independent experiments were analysed with a paired one-sided t test, * p < 0.05, *ns* non significant. D-F) Expression of *ifas-1* is observed in transgenic worms carrying an *ifas-1* transcriptional reporter. D) Representative confocal images with simultaneous visualisation of *ifas-1*p::GFP in green, *myo-2*p::mCherry from the coinjection marker in red, and autofluorescence in white (upper left panel) or only *ifas-1*p::GFP in white (upper right panel; acquired with a spinning disk microscope). GFP expression can be seen in several neurons and in sheath cells in the head (upper panels) and in the tail (lower left panel), in the CAN, and HSN neurons (lower right panel); scale bar, 10 µm, nr, nerve ring, ph, pharynx, dc, dorsal cord, int, intestine, sh, sheath cells, vul, vulva. E) Images of young adult transgenic worms under normal culture conditions (left) or 4 h after wounding (right), where ca. 20% of the worm present a strong induction of *ifas-1*p::GFP in the epidermis; scale bar, 500 µm. F) After 8 h of infection, *ifas-1*p::GFP expression is induced in less than 10% of the population. The increased GFP expression is always seen in the epidermis (epi), as exemplified by the selected worms in the middle panel and one worm shown at higher magnification on the right, compared to control worms (left panel); scale bar, 500 µm in left and middle panels, 100 µm in the right panel.

## Description

The natural fungal pathogen *Drechmeria coniospora* pierces the worm’s cuticle and its hyphae grow throughout the organism. In the epidermis, this triggers a rapid increase in the expression of genes from the *nlp* (for neuro-peptide-like protein) and *cnc* (caenacin) families. These genes encode structurally-related antimicrobial peptides (AMPs). We have defined major signalling pathways required for the regulation of *nlp‑29* gene expression. Two of them, one specific for infection and the second also activated by wounding, act upstream of a highly conserved p38 MAPK signalling cascade. The induction of *cnc‑2* upon infection, on the other hand, is independent of PMK-1/p38 MAPK, but requires DBL-1/TGFß produced by certain neuronal cells, acting via a non-canonical TGFß pathway in epidermal cells. The STAT transcription factor-like protein, STA‑2, is essential for both the PMK-1/p38 MAPK, and DBL-1/TGFß immune signalling pathways, to govern the transcriptional response to fungal infection in the epidermis (reviewed in Kim and Ewbank, 2018; Martineau *et al.*, 2021).

In the absence of infection, expression of a constitutively active Gα protein (GPA-12gf) in the adult epidermis leads to higher expression of AMP genes of both the *nlp* and *cnc* families (Labed *et al.*, 2012). More generally, there is a considerable overlap between the genes induced by infection (Engelmann *et al.*, 2011) and those up-regulated upon expression of GPA-12gf (Lee *et al.*, 2018). To broaden our understanding of the host response to fungal infection, we selected 10 strongly-induced genes for validation through qRT-PCR. We confirmed that for 9 of them, expression was increased in both the infected and the GPA-12gf samples. They included members of the *nlp* and *cnc* families, but also several other genes predicted to encode small secreted peptides, like *F48C1.9* (Omi and Pujol, 2019), *fip-6* (Pujol *et al.*, 2012), *F57H12.6* and *ora-1*. For only one, *F40H7.12*, expression was induced by fungal infection but not in the GPA-12gf background (Figure 1A). The gene encodes a protein with a fascin domain, associated with actin binding. Two other *C. elegans* genes (*F09C6.1* and *Y105C5B.14*) are predicted to encode proteins with a fascin domain (Figure 1B). Data in Wormbase indicates that their expression increases upon exposure to a variety of stresses. We therefore called this family *ifas* for “inducible fascin domain”. We determined that the induction of *F40H7.12*/*ifas-1* upon fungal infection does not require *sta-2,* unlike *nlp-29*, *cnc-2* or *fip-6* (Figure 1C).

We made two different reporter transgenes to study the gene’s expression pattern, both containing the short (800 bp) intergenic region separating *ifas-1* from its upstream gene, one with the 3’ UTR of *unc-54*, the other with its own 3’ UTR. Transgenic strains produced with the two constructs behaved similarly. A constitutive expression was observed in a subset of neurons including neurons in the head, lateral neurons, including the CAN neurons, the HSN neurons and a subset of retrovesicular ganglion neurons (Figure 1D). Upon fungal infection and wounding, an induction was observed in the epidermis. While the induction was robust and reproducible, it was only observed in less than 20% of the worms (Figure 1E-F). This may be because the reporter constructs do not contain all the regulatory elements required to reflect endogenous gene expression. Future characterization of *ifas-1* is expected to reveal previously unexplored aspects of the innate immune response to epidermal fungal infection.

## Methods

Multiple Sequence Alignments of IFAS-1 (CE38709), IFAS-2 (CE15760) and IFAS-3 (CE24063) were done with MUSCLE https://www.ebi.ac.uk/Tools/msa/muscle/ and shaded with Boxshade https://embnet.vital-it.ch/software/BOX_form.html. Protein sequences correspond to those from WormBase release WS280.

Strains: All *C. elegans* strains were maintained on nematode growth medium (NGM) and fed with *E. coli* OP50, as described (Stiernagle, 2006): the wild-type N2, IG1570 *frSi2[pNP138(col-19p::GPA-12gf), unc-119(+) ttTi5605]* II; *frIs7[nlp-29p::GFP, col-12p::DsRed] IV* (Lee *et al.*, 2018), IG1241 *sta-2(ok1860) V* (Dierking *et al.*, 2011).

Constructs: pNP150 (*ifas-1p::GFP::3’UTRunc-54*) is derived from pCFJ151 that was a gift from Erik Jorgensen (Addgene plasmid # 19330; http://n2t.net/addgene:19330; RRID:Addgene_19330) (Frokjaer-Jensen *et al.*, 2008). pNP150 (*ifas-1p::GFP::3’UTRunc-54*) was obtained by Gibson fusion of 800 bp of the *ifas-1* promoter with primers cgactcactagtgggcagcctcaaaatactggatcac and gttcttctcctttactcatagcgttgcccatcagaaac. pNP153 (*ifas-1p::GFP::3’UTRifas-1*) was obtained by replacing the *unc-54* 3’UTR in pNP150 by 250 bp of the *ifas-1* 3’UTR with by Gibson fusion with primers ggatgaactatacaaatagtggtgatccatatttgtaag and gagaatgtctagaactaggcacccaacaaagttagctagc. Each construct was independently injected at 20 ng/µl together with pCFJ90 *myo-2p::mCherry* at 2 ng/µl, pZX13 at 20 ng/µl, pBSKS empty vector at 60 ng/µl. pCFJ90 was a gift from Erik Jorgensen (Addgene plasmid # 19327; http://n2t.net/addgene:19327; RRID:Addgene_19327) (Frokjaer-Jensen *et al.*, 2008), pZX13 contains the hygromycin resistance gene HygR under the control of a minimal 380 bp *rsp-0* promoter sequence (atttttgctttcgtcgtaaa to aatatgtcaggcggtgccgc). It was derived from SG120 (a kind gift of Jason Chin; Radman *et al.*, 2013) by removing the *B0393.2* gene, to decrease lethality associated with the original plasmid (S. O. unpublished observations). Two independent transgenic strains were obtained IG2065 *frEx646[pNP150(ifas-1p::GFP::3’UTRunc-54), myo-2p::mCherry, rps-0p::HygR*] and IG2066 *frEx647[pNP153(ifas-1p::GFP::3’UTRifas-1), myo-2p::mCherry, rps-0p::HygR*]. Images for IG2065 are presented in Figure 1 D-F.

Images were taken of worms mounted on a 2% agarose pad on a glass slide, anesthetized with 0.25 mM levamisole, using either a Leica MZ16 F stereomicroscope, a Zeiss LSM780 confocal microscope or a Visitron spinning disk, as previously described (Taffoni *et al.*, 2020).

Infection & qRT-PCR: Worms were synchronised by the standard bleach method and exposed to fungal spores for 8 h at the L4 stage, or wounded with a microinjection needle at the young adult stage, as previously described (Pujol *et al.*, 2008). RNA extraction and qRT-PCR were done with transcript specific primers, as previously described (Pujol *et al.*, 2008); 3 replicates were analysed.

## Reagents

qRT PCR primers:

**Table:** 

name	gene	sequence	WormBase associated Gene ID
1087	cnc-1 F	CTGCGCAATGGGGATATAACTCA	WBGene00000555
1088	cnc-1 R	GAGAAGACCACCTCCACCAT	WBGene00000555
944	cnc-2 F	CCGCTCAATATGGTTATGGAG	WBGene00000556
549	cnc-2 R	TCCCATGCCCATACCGTAAC	WBGene00000556
1124	cnc-4 F	ACAATGGGGCTACGGTCCATAT	WBGene00000558
1125	cnc-4 R	ACTTTCCAATGAGCATTCCGAGGA	WBGene00000558
2340	ifas-1 F	TTCCTGAGTGCTCACGAAGG	WBGene00044379
2341	ifas-1 R	AACACTGAGGAACGACCAGG	WBGene00044379
2189	F48C1.9 F	CCAATTAAGTACAGCTGCAA	WBGene00018601
2190	F48C1.9 R	GTATCCAGGATAACTGTAATAG	WBGene00018601
2338	F57H12.6 F	GGAAGAAGATCTCCACCTTG	WBGene00019021
2339	F57H12.6 R	AATCGATAACTTCACGAGTC	WBGene00019021
2328	fip-6 F	TGCAATTGTAACATACGCAC	WBGene00009964
2590	fip-6 R	TAATATGGTTGATATCCACC	WBGene00009964
952	nlp-29 F	TATGGAAGAGGATATGGAGGATATG	WBGene00003767
848	nlp-29 R	TCCATGTATTTACTTTCCCCATCC	WBGene00003767
969	nlp-34 F	ATATGGATACCGCCCGTACG	WBGene00015046
970	nlp-34 R	CTATTTTCCCCATCCGTATCC	WBGene00015046
2336	ora-1 F	CAAAGACAAGGAATCGAAGC	WBGene00003879
2337	ora-1 R	TCATCCTTCACGTTCTCATC	WBGene00003879
